# Ectopic Pancreatic Tissue in the Cecum

**DOI:** 10.4103/1319-3767.65179

**Published:** 2010-07

**Authors:** Vipul D. Yagnik, Keyuri B. Patel, Paresh A. Patel, Faruq I. Mulla

**Affiliations:** Department of Surgery, Pramukhswami Medical College, Karamsad-388 325, Gujarat, India. E-mail: vipul_yagnik@yahoo.com; 1 Department of Pathology, Pramukhswami Medical College, Karamsad-388 325, Gujarat, India

**Figure 1:**
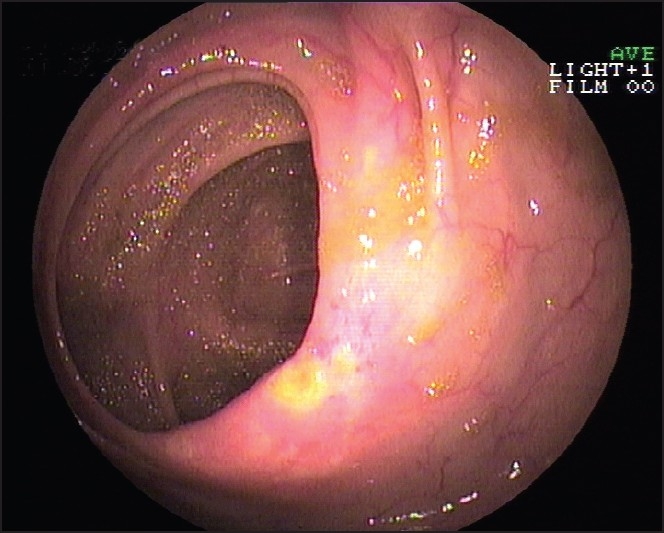
Ulcer in the cecum

**Figure 2:**
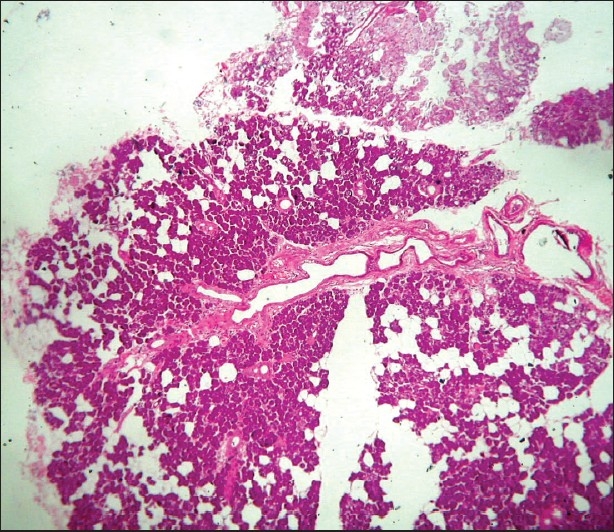
Pancreatic acini and duct system (Hematoxylin and eosin stain, ×100)

Sir,

A 58 years male was admitted to our hospital because of persistent right lower quadrant abdominal pain and melena. He had no history of a similar type of abdominal pain or melena. All blood investigations were normal. Skiagram and USG abdomen were also normal. Stool occult blood was positive. Colonoscopy revealed thickening and inflammation with ulceration in the cecum, no evidence of sub-mucosal nodule with central umbilication was seen [Figure 1]. HPE examination showed pancreatic acini and duct system. Each acinus is made up of irregular clusters of secreting cells. Intercalated ducts are lined by simple low cuboidal epithelium [Figure 2]. After initial medical management, the patient was symptom free. The patient refused surgery. To our knowledge, this is the first case of ectopic pancreatic tissue in the cecum.

Ectopic pancreatic tissue is common and functional. The frequency in autopsy has been reported from 0.55 to 13.7%.[1] The most common sites are the wall of the stomach, duodenum, followed by jejunum, Meckels, and ileum.[2] Most patients with ectopic pancreas are asymptomatic and are diagnosed at endoscopy, operation or autopsy, and, if present, symptoms are non-specific and depend on the location and complications of heterotopic tissue.[3] Typically, heterotopic pancreatic tissue is a sub-mucosal nodule with central umbilication. The diagnosis of ectopic pancreas could be made either by endoscopy or radiological investigation. The definitive diagnosis is made by histopathologic examination. Histopathologic examination reveals normal-appearing pancreatic acinar cells, glands, and sometimes islets of Langerhans. Ectopic pancreatic tissue may cause pain, mucosal bleeding and may sometimes be a lead point for intussusception. Treatment of symptomatic ectopic pancreatic tissue is surgical excision.
